# CXCL12 expression and the survival of patients with gastric cancer: a meta-analysis

**DOI:** 10.1007/s10238-025-01674-3

**Published:** 2025-06-07

**Authors:** Jinxiu Wen, Bingbing Zheng, Ting Fu

**Affiliations:** Department of Gastrointestinal and Hernia Surgery, Heyuan People’s Hospital, Guangdong Provincial People’s Hospital Heyuan Hospital, No. 733, Wenxiang Road, Yuancheng District, Heyuan, 517000 China

**Keywords:** Gastric cancer, CXCL12, Survival, Progression, Meta-analysis

## Abstract

Gastric cancer (GC) remains a leading cause of cancer-related mortality worldwide. CXCL12, a chemokine involved in tumor progression and metastasis, has been inconsistently associated with GC survival. This meta-analysis aimed to evaluate the prognostic significance of CXCL12 expression in GC patients. A comprehensive literature search was conducted in PubMed, Embase, and Web of Science. Observational studies assessing tumor CXCL12 expression and survival outcomes in GC patients were included. Hazard ratios (HRs) with 95% confidence intervals (CIs) were pooled using a random-effects model by incorporating heterogeneity. Ten studies comprising 1361 GC patients were included. High CXCL12 expression was significantly associated with poorer overall survival (OS) (HR: 1.85, 95% CI 1.51–2.26, *p* < 0.001) with mild heterogeneity (*I*^2^ = 17%). Subgroup analyses revealed that the association between high CXCL12 expression and OS was stronger in studies defining high expression as above the median density value (HR: 2.63, 95% CI 1.79–3.86) than in those using any positive expression (HR: 1.61, 95% CI 1.30–2.00; *p* for subgroup difference = 0.03). Additionally, a more pronounced association was observed in studies with follow-up durations ≥ 36 months (HR: 2.42, 95% CI 1.84–3.18) compared to those with < 36 months (HR: 1.59, 95% CI 1.28–1.99; *p* = 0.03). The pooled results also indicated an association between high CXCL12 expression and worse progression-free survival (PFS) (HR: 1.52, 95% CI 1.05–2.20, *p* = 0.03). High CXCL12 expression is associated with poorer survival outcomes in GC patients.

**Supplementary Information:**

The online version contains supplementary material available at 10.1007/s10238-025-01674-3.

## Introduction

Gastric cancer (GC) remains one of the most prevalent and deadly cancers worldwide, despite advances in medical and surgical treatments [1, 2]. It ranks as the fifth most common malignancy and the third leading cause of cancer-related deaths globally [2]. The prognosis for patients with GC is generally poor, largely due to late-stage diagnosis and high rates of metastasis [3]. Understanding the molecular mechanisms underlying GC progression and identifying reliable prognostic biomarkers is essential for improving patient outcomes [4]. CXCL12, also known as stromal cell-derived factor-1 (SDF-1), is a chemokine involved in various physiological and pathological processes, including cell migration, inflammation, and tumor progression [5, 6]. CXCL12 exerts its effects primarily through its receptor CXCR4, a G-protein-coupled receptor expressed on various cell types, including cancer cells [7]. The CXCL12/CXCR4 axis has been implicated in the regulation of cancer cell proliferation, invasion, angiogenesis, and metastasis in several types of cancers [8], including GC [9, 10].

Previous studies have investigated the relationship between CXCL12 expression and the survival of patients with GC, yielding conflicting results [11–20]. Some studies have reported that high CXCL12 expression is associated with poor prognosis [11, 16–20], while others have found no significant association between a high tumor expression of GC and survival [12–15]. These discrepancies may be attributed to differences in study populations, methodologies, and sample sizes, highlighting the need for a comprehensive analysis to clarify the prognostic value of CXCL12 in GC. In view of the uncertainty, we performed meta-analysis aiming to systematically review and synthesize the available evidence on the association between CXCL12 expression and the survival outcomes of patients with GC. By pooling data from multiple studies, we sought to determine the overall impact of CXCL12 expression on overall survival (OS) and progression-free survival (PFS) in this patient population. Additionally, we aimed to explore potential sources of heterogeneity and assess the quality of the included studies to provide robust and clinically relevant conclusions.

## Methods

The study adhered to PRISMA 2020 [21, 22] and the Cochrane Handbook for Systematic Reviews and Meta-analyses [23] guidelines for conducting this meta-analysis, including for the study protocol design, data extraction, statistical analysis, and results presentation. The protocol of the meta-analysis has been prospectively registered at PROSPERO with the identifier: CRD420251003138.

### Literature search

To identify studies pertinent to this meta-analysis, we searched PubMed, Embase, and Web of Science databases using an extensive array of search terms, which included: (1) "CXCL12" OR "SDF1"; (2) "gastric" OR "stomach"; and (3) "cancer" OR "carcinoma" OR "adenoma" OR "adenocarcinoma" OR "malignancy" OR "tumor" OR "tumor" OR "neoplasm". The search was restricted to studies conducted on human subjects and included only full-length articles published in English in peer-reviewed journals. We excluded gray literature such as conference abstracts, dissertations, and preprints to ensure the inclusion of studies with complete methodological details and results that have undergone peer review, thereby enhancing the quality and reliability of the synthesized evidence. Additionally, the references of relevant original and review articles were manually screened to identify any additional eligible studies. The literature search covered the period from the inception of the databases to January 22, 2025. The detailed search strategy for each database is shown in Supplemental File 1.

### Inclusion and exclusion criteria

The inclusion criteria for potential studies were defined according to the PICOS framework:

P (patients): Adult patients (aged 18 years or older) with confirmed diagnosis of GC, regardless of the cancer etiology, stage, or main anticancer treatments.

I (exposure): GC with a high expression of CXCL12. The methods, parameters, and cutoffs for the evaluation of tumor expression of CXCL12 were consistent with those used in the original studies.

C (comparison): Patients with a low tumor expression of CXCL12.

O (outcome): Survival outcomes, including OS and PFS, compared between patients with a high versus a low expression of CXCL12. In general, OS is defined as the time from treatment initiation to death from any cause, while PFS is defined as the time from treatment initiation to disease progression or death, whichever occurs first.

S (study design): Observational studies with longitudinal follow-up, such as cohort studies, nested case–control studies, or post hoc analyses of clinical trials;

Studies were excluded if they were reviews, editorials, meta-analyses, preclinical research, or involved patients without GC, lacked evaluation of tumor expression of CXCL12 as exposure, or did not report the survival outcomes of interest. For studies with overlapping populations, the one with the largest sample size was included in the meta-analysis.

### Study quality assessment and data extraction

The literature search, study selection, quality assessment, and data extraction were independently performed by two authors, with discrepancies resolved through discussion with the corresponding author. Study quality was assessed using the Newcastle–Ottawa Scale (NOS) [24], which evaluates selection, control of confounding factors, and outcome measurement and analysis, with scores ranging from 1 to 9, where a score of 9 indicates the highest quality. Studies with the NOS scores of 7 or above were generally considered as high-quality studies [24]. Data extracted for analysis included study characteristics (author, year, country, and design), participant details (number of patients, mean age, sex, and tumor stage), methods for measuring tumor expression of CXCL12, criteria for defining GC with higher CXCL12 expression, numbers of patients with high CXCL12 expression, median follow-up durations, outcomes reported, and analytic models (univariate or multivariate) for evaluating the association between tumor expression of CXCL12 and survival outcomes of GC.

### Statistical analyses

The associations between tumor expression of CXCL12 and OS/PFS of patients with GC were summarized as hazard ratios (HRs) and corresponding 95% confidence intervals (CIs), compared between patients with a high versus a low expression of CXCL12. The HRs and their standard errors were derived from 95% CIs or *p* values and subsequently log-transformed to stabilize variance and achieve a normalized distribution [23]. To assess heterogeneity, we used the Cochrane *Q* test and *I*^2^ statistics [25], with *I*^2^ < 25%, 25–75%, and > 75% indicating low, moderate, and high heterogeneity. A random-effects model was applied to integrate the results, accounting for study variability [23]. Via excluding individual studies sequentially, a sensitivity analysis was performed to evaluate the robustness of the findings. In addition, subgroup analyses were performed to evaluate study characteristics on the outcomes, such as mean ages of the patients, proportions of men, definitions of a high tumor expression of CXCL12, median follow-up durations, analytic models, and NOS scores. These subgroups were predefined, and continuous variables were dichotomized using the median values across the included studies to minimize data-driven bias. Publication bias was evaluated using funnel plots and visual inspection for asymmetry, supplemented by Egger’s regression test [26]. Analyses were performed using RevMan (Version 5.1; Cochrane Collaboration, Oxford, UK) and Stata software (version 12.0; Stata Corporation, College Station, TX, USA).

## Results

### Study identification

**Fig. 1:**
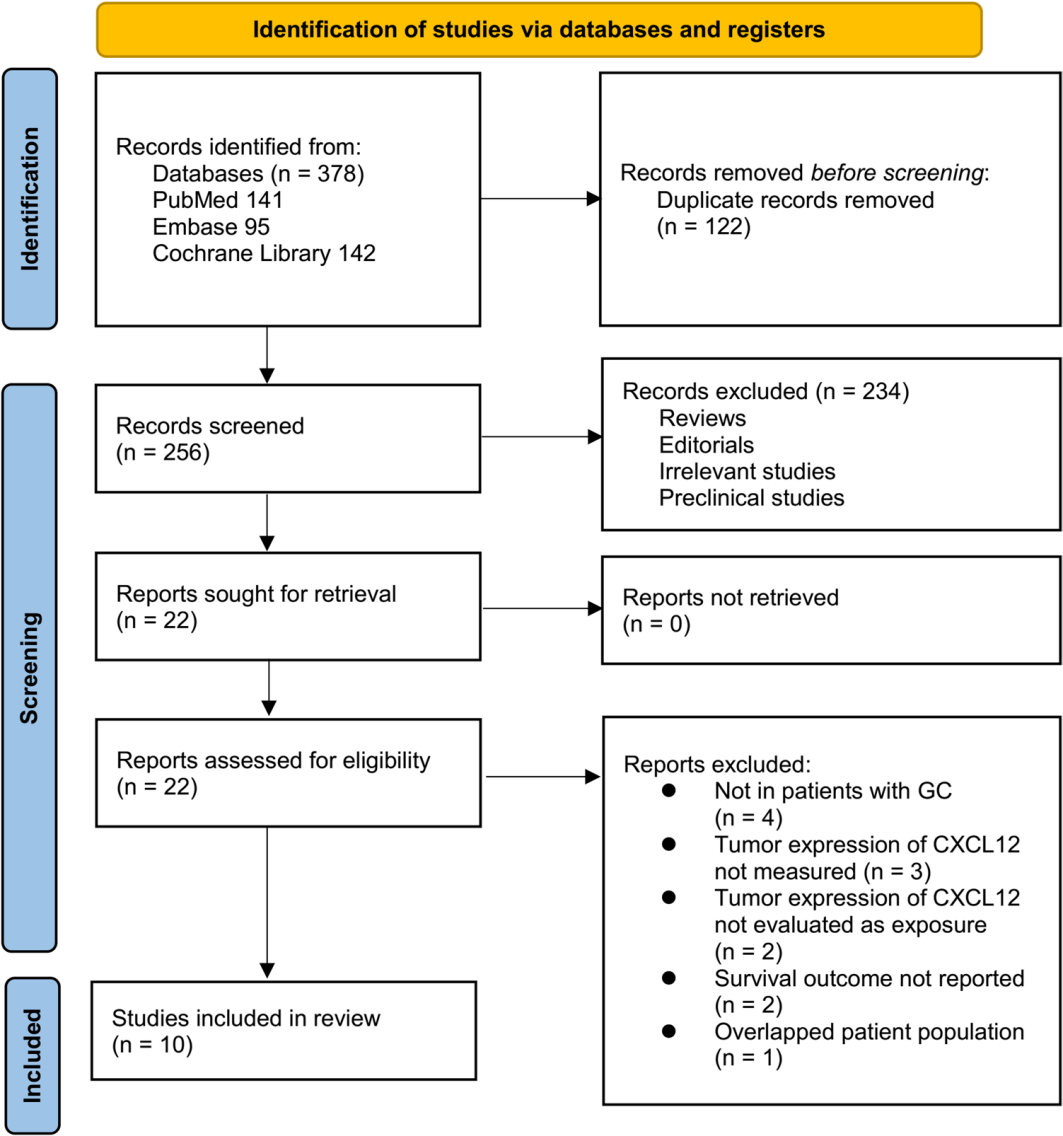
Flowchart of database search and study inclusion

The study selection process is summarized in Fig. 1. A total of 378 potentially relevant records were initially identified from the three databases searched and screening of citations of related articles, with 122 duplicates removed. Screening of titles and abstracts resulted in the exclusion of 234 articles that did not meet the objectives of the meta-analysis. The full texts of the remaining 22 articles were independently reviewed by two authors, leading to the exclusion of 12 studies for various reasons detailed in Fig. 1. Ultimately, ten studies were included in the quantitative analysis [11–20].

### Overview of the study characteristics

Table 1 shows the summarized characteristics of the available studies included in the meta-analysis. Overall, nine retrospective [11, 13–20] and one prospective cohort [12] studies were identified. These studies were published between 2007 and 2024, and performed in Japan, Germany, and China. Patients with GC undergoing surgical resection were included in nine studies [11–17, 19, 20], while patients with advanced GC receiving immune checkpoint inhibitors were included in the other study [18]. A total of 1361 patients with GC were included in these studies. The mean ages of the patients varied from 56.0 to 69.5 years, and the proportion of men varied from 54.0 to 74.8%. Tumor expression of CXCL12 was evaluated with immunohistochemistry (IHC) in all studies except one study, in which reverse transcription-polymerase chain reaction (RT-PCR) was used to evaluated the expression of CXCL12 [12]. A high tumor expression of CXCL12 was defined as the positive tumor expression of CXCL12 in five studies [11–14, 17], the presence of CXCL12 in > 5% [19] or > 30% [15] of cancer cells in two studies, and the presence of CXCL12 above median density value in another three studies [16, 18, 20]. Accordingly, 767 (56.4%) patients had high tumor expression of CXCL12. The median follow-up durations were 10 to 79 months. The outcome of OS was reported in all the included studies [11–20], while the outcome of PFS was reported in two studies [13, 17]. The association between tumor expression of CXCL12 and the survival outcomes of patients with GC was evaluated using univariate analysis in six studies [13–15, 17, 18, 20], and with multivariate analysis in four studies [11, 12, 16, 19]. The included studies achieved NOS scores ranging from five to nine, reflecting a generally moderate to high quality of methodology and reporting (Table 2).

**Table 1:** Characteristics of the included cohort studies

Study	Country	Study design	Diagnosis	No. of patients	Mean age (years)	Male (%)	Tumor stage	Methods for measuring CXCL12	Criteria of higher CXCL12 expression	No. of patients with high CXCL12 expression	Median follow-up duration (months)	Outcome reported	Analytic models
Ishigami 2007	Japan	RC	GC patients undergoing curative gastrectomy	185	61	71.4	I–IV	IHC	Positive tumor expression of CXCL12	78	30	OS	Multivariate
Schimanski 2011	Germany	PC	Patients undergoing surgery for esophagogastric adenoma	69	66.6	62	I–IV	RT-PCR	Positive tumor expression of CXCL12	57	33	OS	Multivariate
Ying 2012	China	RC	GC patients undergoing curative gastrectomy	50	56	68	I–IV	IHC	Positive tumor expression of CXCL12	45	71	OS and PFS	Univariate
Masuda 2014	Japan	RC	GC patients undergoing gastrectomy	111	62.9	74.8	I–IV	IHC	Positive tumor expression of CXCL12	98	43	OS	Univariate
Satomura 2014	Japan	RC	GC patients undergoing gastrectomy	137	68.9	74.5	I–IV	IHC	Presence of CXCL12 in > 30% of cancer cells	43	79	OS	Univariate
Wang 2014	China	RC	GC patients undergoing gastrectomy	180	NR	68.9	I–IV	IHC	Presence of CXCL12 above median density value	132	70	OS	Multivariate
Izumi 2016	Japan	RC	GC patients undergoing gastrectomy	110	69.5	71.8	NR	IHC	Positive tumor expression of CXCL12	64	33	OS and PFS	Univariate
Qin 2021	China	RC	GC patients undergoing gastrectomy	285	NR	54	I–IV	IHC	Presence of CXCL12 in > 5% of cancer cells	136	21	OS	Multivariate
Chen 2021	China	RC	Advanced GC patients undergoing ICIs	126	NR	NR	IV	IHC	Presence of CXCL12 above median density value	63	10	OS	Univariate
Wang 2024	China	RC	GC patients undergoing gastrectomy	108	NR	NR	I–IV	IHC	Presence of CXCL12 above median density value	51	48	OS	Univariate

CXCL12, C-X-C motif chemokine ligand 12; GC, gastric cancer; IHC, immunohistochemistry; ICIs, immune checkpoint inhibitors; NR, not reported; OS, overall survival; PC, prospective cohort; PFS, progression-free survival; RC, retrospective cohort; RT-PCR, reverse transcription-polymerase chain reaction

**Table 2:** Study quality evaluation via the Newcastle–Ottawa Scale

Ishigami 2007	Representativeness of the exposed cohort	Selection of the non-exposed cohort	Ascertainment of exposure	Outcome not present at baseline	Control for age and sex	Control for other confounding factors	Assessment of outcome	Enough long follow-up duration	Adequacy of follow-up of cohorts	Total
Ishigami 2007	0	1	1	1	1	1	1	1	1	8
Schimanski 2011	1	1	1	1	1	1	1	1	1	9
Ying 2012	0	1	1	1	0	0	1	1	1	6
Masuda 2014	0	1	1	1	0	0	1	1	1	6
Satomura 2014	1	1	1	1	0	0	1	1	1	7
Wang 2014	1	1	1	1	1	1	1	1	1	9
Izumi 2016	1	1	1	1	0	0	1	1	1	7
Qin 2021	0	1	1	1	1	1	1	0	1	7
Chen 2021	0	1	1	1	0	0	1	0	1	5
Wang 2024	0	1	1	1	0	0	1	1	1	6

### Association between tumor expression of CXCL12 and survival of GC

**Fig. 2:**
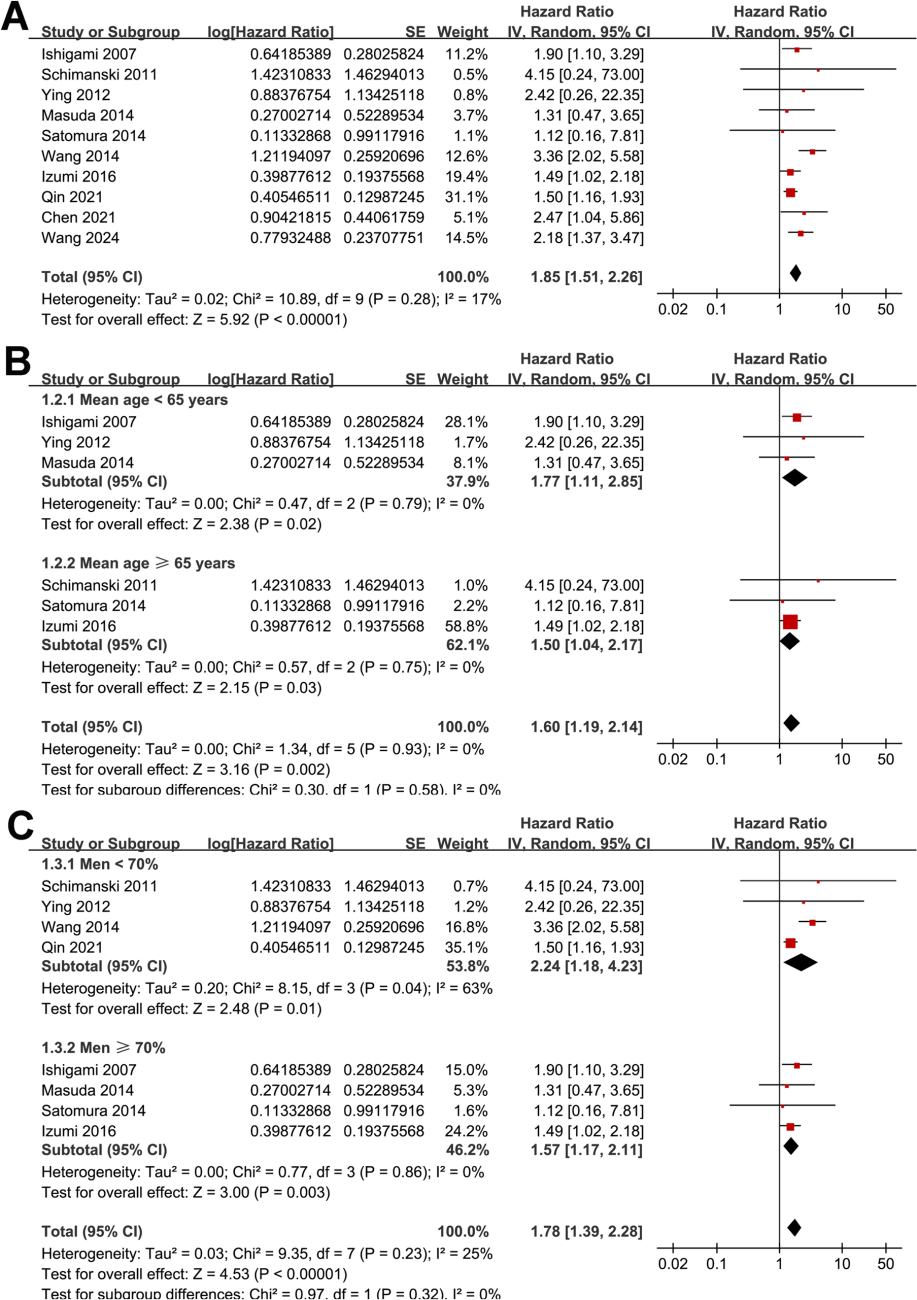
Forest plots for the meta-analyses of the association between tumor expression of CXCL12 and OS of patients with GC; **A** forest plots for the overall meta-analysis of OS; **B** forest plots for the subgroup analysis according to mean ages of the patients; and **C** forest plots for the subgroup analysis according to proportion of men

**Fig. 3:**
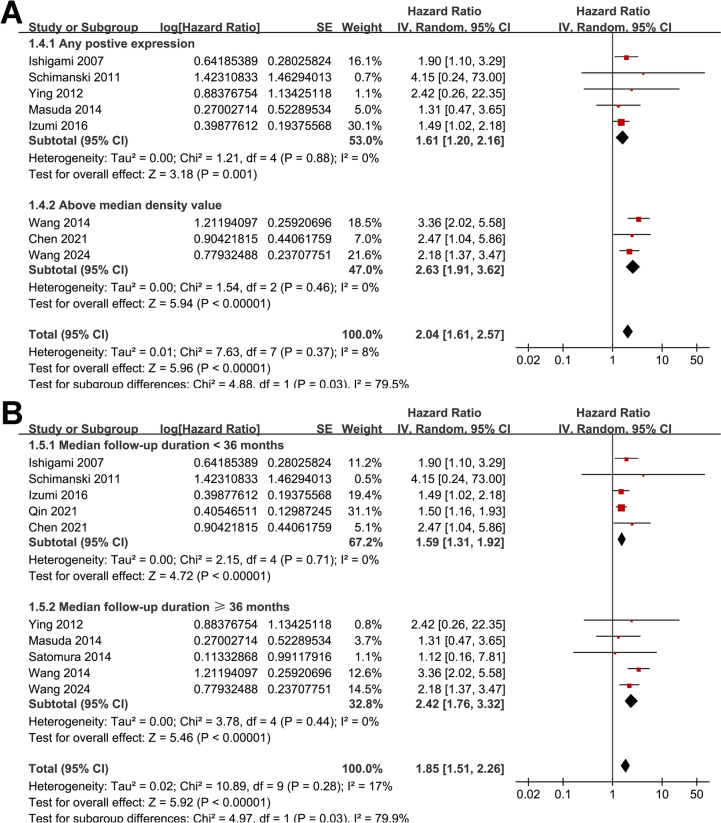
Forest plots for the subgroup analyses of the association between tumor expression of CXCL12 and OS of patients with GC; **A** forest plots for the subgroup analysis according to the definition of a high tumor expression of CXCL12; and **B** forest plots for the subgroup analysis according to the median follow-up durations

**Fig. 4:**
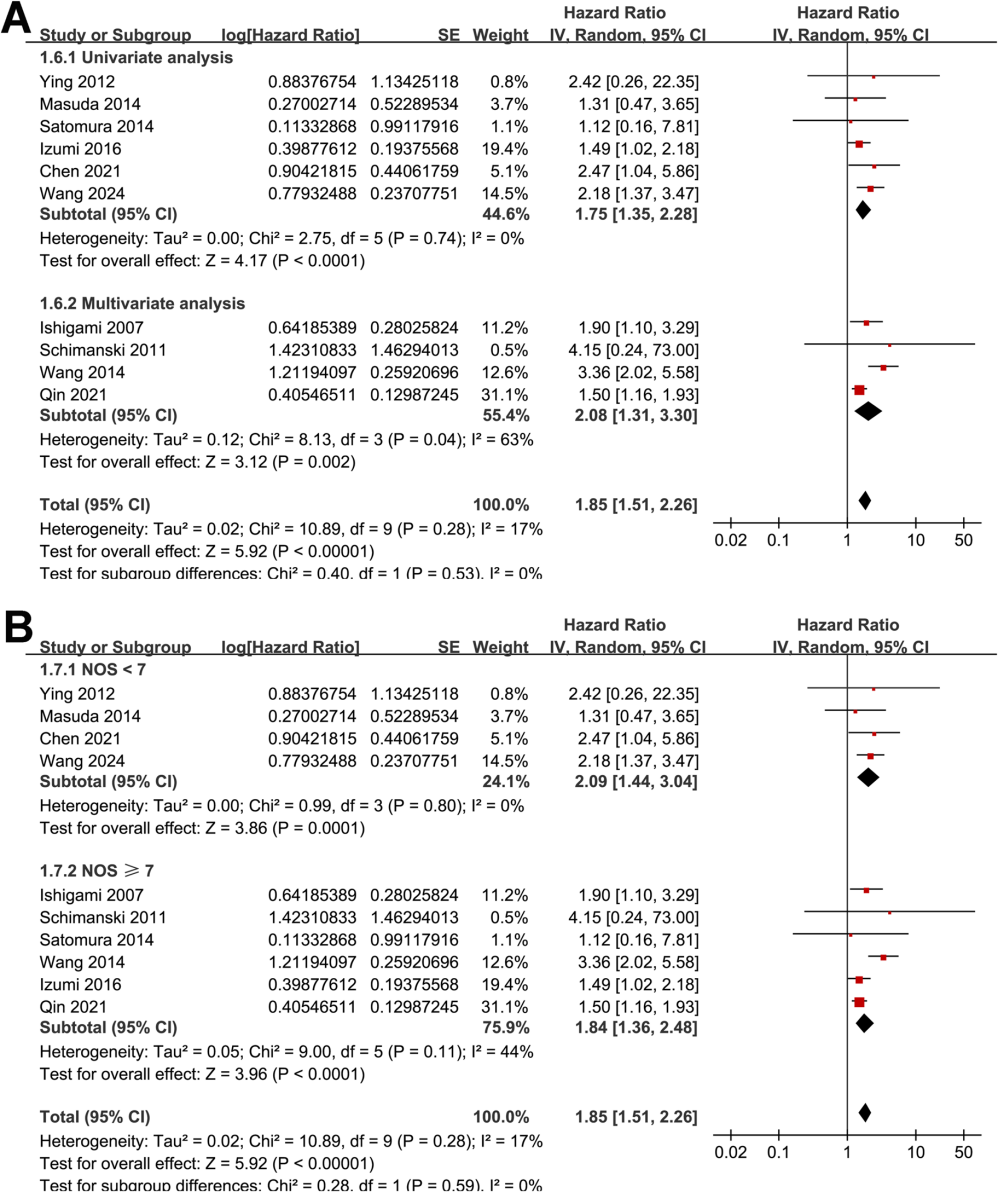
Forest plots for the subgroup analyses of the association between tumor expression of CXCL12 and OS of patients with GC; **A** forest plots for the subgroup analysis according to the analytic models; and **B** forest plots for the subgroup analysis according to the NOS scores

**Fig. 5:**
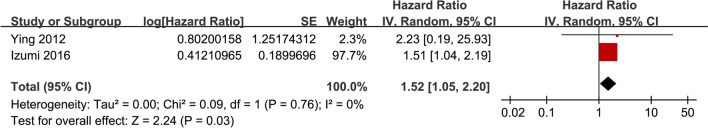
Forest plots for the meta-analyses of the association between tumor expression of CXCL12 and PFS of patients with GC

Overall, ten studies [11–20] evaluated the association between tumor expression of CXCL12 and OS in patients with GC. A mild heterogeneity was observed among these studies (*p* for Cochrane *Q* test = 0.28; *I*^2^ = 17%). The pooled results showed that overall, a high tumor expression of CXCL12 was associated with poorer OS in patients with GC (HR: 1.85, 95% CI 1.51–2.26, *p* < 0.001; Fig. 2A). Sensitivity analyses by excluding one study at a time showed similar results (HR: 1.65–2.00, *p* all < 0.05; Table 3). Specifically, excluding the only study with prospective design and RT-PCR for evaluating tumor CXCL12 expression [12] showed consistent result (HR: 1.85, 95% CI 1.49–2.29, *p* < 0.001; *I*^2^ = 24%). In addition, the subgroup analysis showed that the association between tumor expression of CXCL12 and OS was not significantly affected by the mean age and the proportion of men of the included patients (*p* for subgroup difference = 0.58 and 0.32, Fig. 2B, C). Interestingly, we found that the association between tumor expression of CXCL12 and OS was stronger in studies with a high CXCL12 expression defined as above the median density value as compared to that as any positive expression of CXCL12 (HR: 2.63 vs. 1.61, *p* for subgroup difference = 0.03; Fig. 3A). Moreover, a stronger association between high expression of CXCL12 and OS was also observed in studies with follow-up duration ≥ 36 months as compared to those < 36 months (HR: 2.42 vs. 1.59, *p* for subgroup difference = 0.03; Fig. 3B). Finally, consistent results were obtained in subgroups of studies with univariate and multivariate analyses (*p* for subgroup difference = 0.53, Fig. 4A), and in subgroups of studies with the NOS scores < or ≥ 7 (*p* for subgroup difference = 0.59, Fig. 4B). The pooled results of two studies [13, 17] showed that a high tumor expression of CXCL12 was associated with poorer PFS in patients with GC (HR: 1.52, 95% CI 1.05–2.20, *p* = 0.03; Fig. 5) with no significant heterogeneity (*p* for Cochrane *Q* test = 0.76; *I*^2^ = 0%).

**Table 3:** Results of sensitivity analysis for the outcome of OS

Study excluded	HR (95% CI)	*P* for overall effect	*I*^2^ (%)
Ishigami 2007	1.87 [1.47, 2.37]	< 0.001	26
Schimanski 2011	1.85 [1.49, 2.29]	< 0.001	24
Ying 2012	1.86 [1.49, 2.31]	< 0.001	26
Masuda 2014	1.89 [1.52, 2.35]	< 0.001	24
Satomura 2014	1.87 [1.51, 2.33]	< 0.001	25
Wang 2014	1.65 [1.39, 1.96]	< 0.001	0
Izumi 2016	1.96 [1.55, 2.49]	< 0.001	19
Qin 2021	2.00 [1.62, 2.49]	< 0.001	0
Chen 2021	1.83 [1.47, 2.27]	< 0.001	22
Wang 2024	1.81 [1.44, 2.28]	< 0.001	20

OS, overall survival; HR, hazard ratio; CI, confidence interval

### Publication bias

**Fig. 6:**
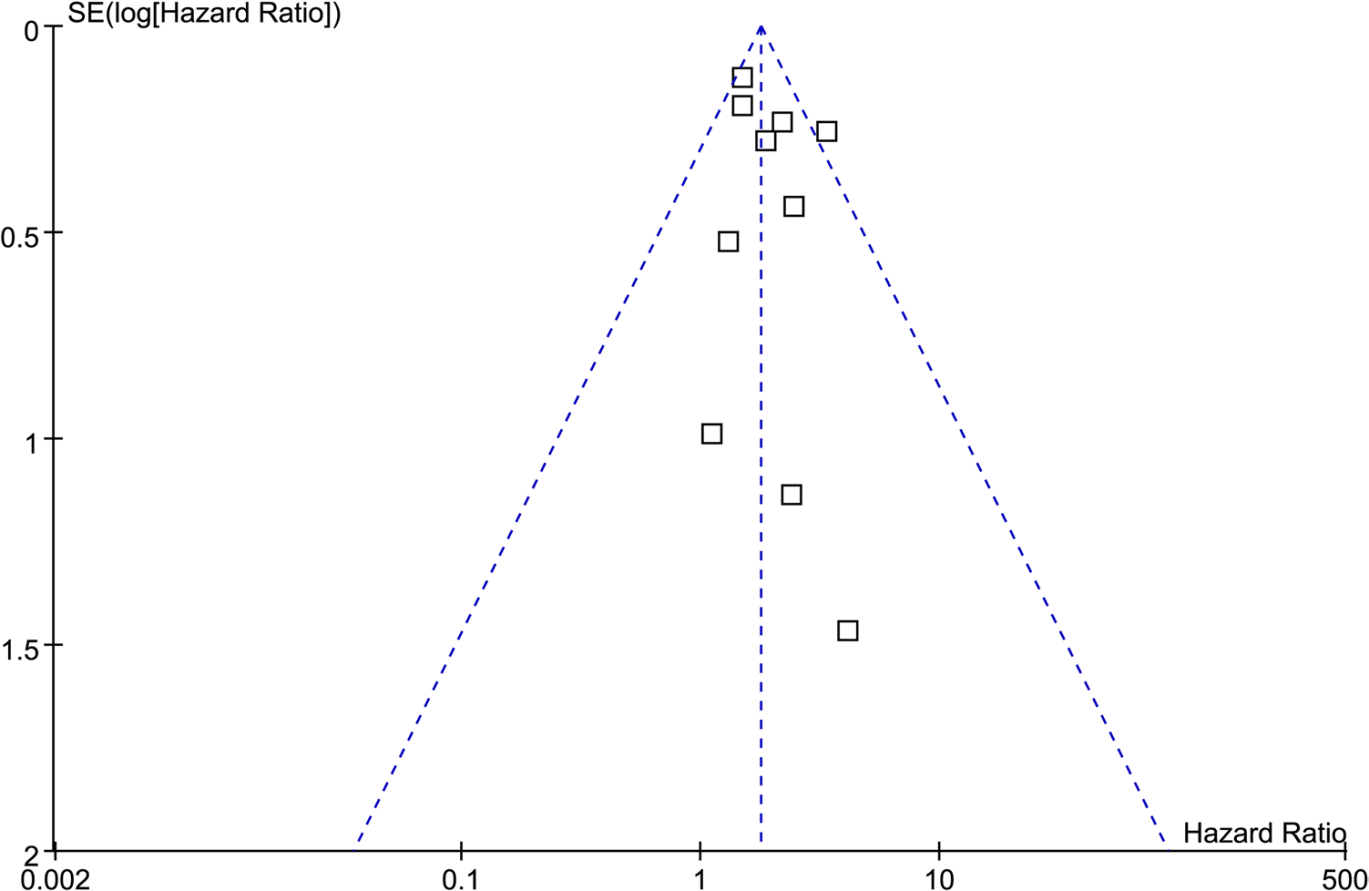
Funnel plots for evaluating the publication bias underlying the meta-analysis of the association between tumor expression of CXCL12 and OS of patients with GC

The funnel plots for the meta-analyses assessing the association between tumor expression of CXCL12 and OS of patients with GC are shown in Fig. 6. Visual inspection of the plots reveals symmetry, indicating a low risk of publication bias. These findings are further supported by Egger’s regression analyses (*p* = 0.55). The publication bias underlying the meta-analysis for the association between tumor expression of CXCL12 and PFS could not be determined because only two studies were available.

## Discussion

This meta-analysis demonstrated that high CXCL12 expression is significantly associated with poorer OS and PFS in patients with GC. By pooling data from ten studies with 1,361 patients, we found that patients with high tumor CXCL12 expression had a significantly higher risk of mortality compared to those with low expression. The association remained robust across sensitivity analyses, and subgroup analyses provided additional insights into potential modifiers of this relationship. These findings highlight CXCL12 as a potential prognostic biomarker for GC and suggest that its role in tumor progression warrants further investigation.

Several molecular mechanisms may underlie the link between high CXCL12 expression and poor survival in GC. CXCL12, primarily acting through its receptor CXCR4, is known to play a pivotal role in tumor growth, metastasis, and immune evasion [27, 28]. The CXCL12/CXCR4 axis promotes epithelial-mesenchymal transition (EMT), enhances tumor cell migration and invasion, and facilitates distant metastasis through interactions with the tumor microenvironment [29]. Additionally, CXCL12 is involved in angiogenesis by recruiting endothelial progenitor cells and increasing vascular endothelial growth factor (VEGF) expression, thereby promoting tumor vascularization [30]. Furthermore, CXCL12 contributes to immune suppression by attracting regulatory T cells (Tregs) and myeloid-derived suppressor cells (MDSCs), which inhibit antitumor immune responses [31, 32]. Given these mechanisms, high CXCL12 expression likely contributes to a more aggressive GC phenotype with greater metastatic potential and resistance to conventional treatments, leading to poorer survival outcomes.

Subgroup analyses provided further insights into factors influencing the prognostic significance of CXCL12 in GC. Notably, the association between high CXCL12 expression and OS was stronger in studies that defined high CXCL12 as above the median density value rather than as any positive expression. This finding suggests a dose–response relationship, where the intensity or extent of CXCL12 expression within tumors may be crucial in determining prognosis. Tumors with higher CXCL12 density may exhibit a more aggressive biological behavior, leading to increased tumor proliferation, invasion, and metastasis [33]. Additionally, we observed a stronger association between high CXCL12 expression and OS in studies with follow-up durations of ≥ 36 months compared to those with shorter follow-ups. This may indicate that the prognostic impact of CXCL12 is more pronounced over the long term, potentially reflecting its role in promoting late-stage metastasis and disease recurrence rather than immediate postoperative outcomes [34]. The prolonged effect of CXCL12 on GC progression could be attributed to its role in creating a tumor-permissive microenvironment, facilitating sustained tumor growth and dissemination over time [35].

This study has several strengths. First, we performed a comprehensive and up-to-date literature search, ensuring the inclusion of all relevant studies published to date. Second, we included only longitudinal studies, strengthening the reliability of the findings by focusing on temporally relevant survival outcomes. Third, multiple sensitivity and subgroup analyses were conducted to validate the robustness of the findings and explore sources of heterogeneity. The consistency of results across different analytical models and patient subgroups enhances the credibility of the association between CXCL12 and GC survival. Despite these strengths, several limitations should be acknowledged. Most of the included studies were retrospective in design, making them susceptible to selection bias and confounding [36]. Although multivariate analyses were conducted in some studies, the potential impact of unmeasured confounders, such as treatment regimens, molecular subtypes of GC, and other tumor microenvironmental factors, cannot be ruled out. Additionally, the number of included studies was relatively limited, particularly for PFS analysis, which was reported in only two studies. This restricts the generalizability of the findings and highlights the need for further research with larger datasets. Moreover, while we observed a significant association between high CXCL12 expression and survival outcomes, these findings do not establish causality due to the observational nature of the included studies. Furthermore, another limitation is the lack of adjustment for key confounding factors. The proportion of patients with high vs. low CXCL12 expression varied widely across studies, and small group sizes in some cohorts may reduce the statistical power and reliability of subgroup comparisons. Additionally, treatment regimens were not consistently reported or stratified by CXCL12 expression status, precluding evaluation of whether treatment effects differed between expression groups. As CXCL12 may influence treatment response—especially to immunotherapy—future studies should collect and report such data in a standardized manner to enable more detailed subgroup analyses and clarify the independent prognostic value of CXCL12. Finally, there is variability in methods used to assess CXCL12 expression and the thresholds for defining high expression. Differences in immunohistochemistry protocols, antibodies, scoring systems, and detection platforms (e.g., RT-PCR vs. IHC) may contribute to heterogeneity. To improve consistency and comparability in future research, it is crucial to adopt standardized detection methodologies and unified scoring systems, such as the H-score [37], for evaluating CXCL12 expression in GC.

It is also important to acknowledge the potential influence of geographic and clinical heterogeneity on our findings. Most included studies originated from East Asian countries (Japan and China), where intestinal-type GC predominates and surgical practices such as D2 lymphadenectomy are standard [38]. In contrast, diffuse-type GC is more common in Western populations (e.g., Germany), where different staging systems and adjuvant treatments may apply [39]. These subtype distributions and treatment strategies can influence both CXCL12 expression patterns and survival outcomes. Moreover, gene-environment interactions—such as regional variations in CXCL12 gene polymorphisms, H. pylori infection, EBV prevalence, and lifestyle factors—may affect chemokine expression and tumor behavior [40, 41]. Additionally, methodological differences, including CXCL12 detection methods (IHC vs. RT-PCR), scoring criteria (e.g., positivity thresholds vs. H-score), and follow-up durations, could introduce bias. Although our pooled analysis showed low heterogeneity, these contextual factors underscore the need for cautious interpretation. Our findings are most generalizable to East Asian and European populations; thus, further validation in underrepresented regions such as North America, Africa, and South America is warranted to enhance global applicability.

The clinical implications of this meta-analysis suggest that CXCL12 could serve as a valuable prognostic biomarker for GC. Assessing tumor CXCL12 expression may help stratify patients based on their risk of disease progression and guide personalized treatment strategies. For instance, patients with high CXCL12 expression may benefit from closer postoperative monitoring and more aggressive therapeutic interventions. However, despite its prognostic potential, targeting CXCL12 therapeutically remains a challenge. While CXCR4 antagonists such as plerixafor have been investigated in preclinical and early-phase clinical studies, their effectiveness in GC remains uncertain [42]. One major hurdle is that CXCL12 plays essential roles in normal physiological processes, including hematopoiesis and immune regulation, raising concerns about potential off-target effects. Additionally, tumor heterogeneity and compensatory signaling pathways may limit the efficacy of CXCL12-targeted therapies, necessitating further investigation into combination strategies with other immunotherapies or targeted treatments. Accordingly, several CXCR4 antagonists, such as plerixafor (AMD3100), BL-8040 (motixafortide), and balixafortide, have been evaluated in early-phase trials for solid tumors. For instance, the CXCR4 antagonist BL-8040 has shown encouraging activity when combined with pembrolizumab and chemotherapy in pancreatic cancer [43], while balixafortide demonstrated synergistic efficacy with eribulin in metastatic breast cancer [44].

In addition, plerixafor has been tested in combination with immune checkpoint inhibitors in patients with advanced pancreatic cancer (NCT04177810). Although promising, these approaches are still in early development, and further trials specifically targeting the CXCL12/CXCR4 axis in GC are warranted. Standardizing patient selection based on CXCL12 expression may also improve trial outcomes and therapeutic efficacy. Future research should focus on standardizing CXCL12 assessment methodologies to improve comparability across studies. Large-scale prospective cohort studies with uniform definitions of high CXCL12 expression are needed to validate its prognostic value. Mechanistic studies should further elucidate the specific pathways through which CXCL12 influences GC progression and resistance to therapy. Additionally, clinical trials evaluating CXCL12/CXCR4-targeted therapies in GC patients with high CXCL12 expression could provide insights into the therapeutic potential of this axis. Investigating how CXCL12 interacts with other components of the tumor microenvironment, such as immune cells and stromal fibroblasts, may also uncover novel therapeutic targets.

## Conclusions

In conclusion, this meta-analysis provides summarized evidence that high CXCL12 expression is associated with poorer survival outcomes in patients with GC. The findings reinforce the role of the CXCL12/CXCR4 axis in GC progression and highlight its potential as a prognostic biomarker. However, the variability in CXCL12 assessment methods and the observational nature of the included studies necessitate cautious interpretation. Further prospective research is required to validate these findings and explore the feasibility of CXCL12-targeted therapies in GC management.

## Supplementary Information

Below is the link to the electronic supplementary material.

Supplementary file1 (DOCX 13 kb)

## Data Availability

The authors confirm that the data supporting the findings of this study are available within the article. Further inquiries can be directed to the corresponding author.
