# Supplementary material for: CXCL12 expression and the survival of patients with gastric cancer: a meta-analysis
Source: Clin Exp Med. 2025 Jun 7;25(1):191. doi: 10.1007/s10238-025-01674-3 (PMC12145318; doi:10.1007/s10238-025-01674-3)
Supplement: Supplementary file 1 — Supplementary file1 (DOCX 13 kb) [file 10238_2025_1674_MOESM1_ESM.docx]

**Search strategy for each database**

**PubMed**

("CXCL12" OR "SDF1" OR "Stromal cell-derived factor 1" OR "SDF-1") AND ("Stomach Neoplasms"[Mesh] OR "gastric cancer" OR "stomach cancer" OR "gastric carcinoma" OR "stomach carcinoma" OR "gastric adenocarcinoma" OR "stomach adenocarcinoma" OR "gastric malignancy" OR "stomach malignancy" OR "gastric tumor" OR "stomach tumor" OR "gastric tumour" OR "stomach tumour" OR "gastric neoplasm" OR "stomach neoplasm")

**Embase**

('cxcl12'/exp OR 'cxcl12' OR 'sdf1' OR 'stromal cell-derived factor 1' OR 'sdf-1') AND ('stomach cancer'/exp OR 'gastric cancer' OR 'stomach cancer' OR 'gastric carcinoma' OR 'stomach carcinoma' OR 'gastric adenocarcinoma' OR 'stomach adenocarcinoma' OR 'gastric malignancy' OR 'stomach malignancy' OR 'gastric tumor' OR 'stomach tumor' OR 'gastric tumour' OR 'stomach tumour' OR 'gastric neoplasm' OR 'stomach neoplasm') AND [humans]/lim AND [clinical study]/lim AND [embase]/lim

**Web of Science**

TS=("CXCL12" OR "SDF1" OR "Stromal cell-derived factor 1" OR "SDF-1") AND TS=("gastric cancer" OR "stomach cancer" OR "gastric carcinoma" OR "stomach carcinoma" OR "gastric adenocarcinoma" OR "stomach adenocarcinoma" OR "gastric malignancy" OR "stomach malignancy" OR "gastric tumor" OR "stomach tumor" OR "gastric tumour" OR "stomach tumour" OR "gastric neoplasm" OR "stomach neoplasm")
